# Alterations of the duodenal mucosal microbiome in patients with metabolic dysfunction-associated steatotic liver disease

**DOI:** 10.1038/s41598-024-59605-3

**Published:** 2024-04-21

**Authors:** Mengting Ren, Hanghai Pan, Xinxin Zhou, Mosang Yu, Feng Ji

**Affiliations:** 1 Department of Gastroenterology, The First Affiliated Hospital, Zhejiang University School of Medicine, 79 Qingchun Road, Hangzhou, 310003 Zhejiang China; 2 Cancer Center, Department of Gastroenterology, Zhejiang Provincial People’s Hospital (Affiliated People’s Hospital), Hangzhou Medical College, 158 Shangtang Road, Hangzhou, 310014 Zhejiang China

**Keywords:** Gut microbiota, Metabolic dysfunction-associated steatotic liver disease, Small intestine, Mucosa-associated microbiota, Biomarkers, Gastroenterology

## Abstract

Metabolic dysfunction-associated steatotic liver disease (MASLD), formerly known as nonalcoholic fatty liver disease (NAFLD), is associated with altered gut microbiota; however, there has been a focus on fecal samples, which are not representative of the entire digestive tract. Mucosal biopsies of the descending duodenum were collected. Five regions of the 16S rRNA gene were amplified and sequenced. Other assessments conducted on the study subjects included body mass index, transient elastography, liver enzymes, and lipid profile. Fifty-one subjects (36 with MASLD and 15 controls) were evaluated. There was no significant difference between the two groups regarding alpha- or beta-diversity of the duodenal mucosal microbiota. Linear discriminant analysis effect size (LEfSe) analysis showed that the genera *Serratia* and *Aggregatibacter* were more abundant in the duodenal mucosa of patients with MASLD, whereas the duodenal mucosal microbiota of the healthy controls was enriched with the genus *Petrobacter*. PICRUSt2 analysis revealed that genes associated with amino acid degradation and carboxylate degradation were significantly enriched in the duodenal mucosal microbiota of patients with MASLD. Our findings reveal the duodenal mucosal microbiota in patients with MASLD, which could contribute to future studies investigating the causal relationship between duodenal microbiota and MASLD.

## Introduction

Metabolic dysfunction-associated steatotic liver disease (MASLD), formerly known as nonalcoholic fatty liver disease (NAFLD), is characterized by excessive lipid deposition in hepatocytes, and is the most common chronic liver disease worldwide with a global prevalence of 25%^1,2^. The spectrum of MASLD ranges from simple steatosis to metabolic dysfunction-associated steatohepatitis (MASH), and the latter can potentially evolve into fibrosis, cirrhosis, and finally into hepatocellular carcinoma^3^. Despite extensive research, the molecular pathogenesis of MASLD has yet to be elucidated^3,4^. There is increasing evidence that gut microbiota dysbiosis might play a vital role in the pathogenesis of MASLD^5,6^. However, most of the gut microbiome studies conducted to date, including those on MASLD^7–9^, have used fecal analysis to investigate the microbiota, primarily because it is easy to sample. Differences in pH, oxygen concentrations, and intestinal motility throughout the gut mean feces may not be representative of the entire intestinal microbiome^10^.

In addition to serving as the primary organ of digestion and absorption, the small intestine also functions as an immune organ and is increasingly implicated in essential microbe-host crosstalk^11,12^. On the one hand, bile produced by the liver is secreted into the duodenum, and dysregulation of hepatic bile production and bile acid metabolism has been reported in patients with MASLD, which may further directly affect the duodenal microbiota^13^. On the other hand, the duodenum drains into the portal vein, so it is reasonable to assume that the duodenal mucosal microbiota exert an important impact on the liver. Compared with luminal microbiota, mucosal microbiota are more likely to translocate across the intestinal barrier^14^. Previous studies have described dysbiosis of the duodenal microbiota in liver diseases including cirrhosis^15–17^, alcoholic liver disease^18^, and chronic liver disease^19^. However, no study to date has characterized the duodenal microbiome in patients with MASLD.

The aim of this study was to investigate the duodenal mucosal microbiome in patients with MASLD in comparison with a healthy control group.

## Results

### Study population and sequencing data

A total of 51 subjects (36 patients with MASLD and 15 healthy controls) were recruited into the study. Demographic and clinical characteristics are summarized in Table 1. There were no significant differences between the two groups in terms of age, gender distribution, smoking status, proton pump inhibitor (PPI) use, metformin use, *H. pylori* infection, and fasting blood glucose levels. However, the MASLD group had significantly higher body mass index (BMI) (*p* < 0.001), alanine transaminase (ALT) (*p* < 0.01), gamma-glutamyl transferase (GGT) (*p* < 0.001), and triglycerides (*p* < 0.05), and lower HDL-cholesterol (*p* < 0.001) compared with healthy controls. In addition, the MASLD group had a mean value of 310.6 ± 23.7 dB/m for controlled attenuation parameter (CAP) and a mean value of 7.6 ± 2.7 kPa for liver stiffness measurement (LSM).

**Table 1:** Demographic and clinical characteristics.

Variable	Healthy controls (*n* = 15)	MASLD (*n* = 36)	*p*-value
Age, years	38.9 ± 13.4	34.2 ± 9.3	0.157
Female, *n* (%)	10 (66.7%)	21 (58.3%)	0.579
BMI, kg/m^2^	23.3 ± 4.1	32.0 ± 4.3	< 0.001
Current smoker, *n* (%)	2 (13.3%)	5 (13.9%)	1.000
PPI user, *n* (%)	2 (13.3%)	2 (5.6%)	0.347
Metformin user, n (%)	1 (6.7%)	1 (2.8%)	0.515
*H. pylori* infection, *n* (%)	4 (26.7%)	9 (25.0%)	1.000
ALT, U/L	15.8 ± 7.6	52.7 ± 48.3	0.007
AST, U/L	18.3 ± 4.8	31.4 ± 25.5	0.063
GGT, U/L	15.1 ± 8.1	51.1 ± 34.1	< 0.001
Triglycerides, mmol/L	1.1 ± 0.3	2.1 ± 1.4	0.035
Total cholesterol, mmol/L	4.5 ± 0.7	4.6 ± 0.8	0.834
LDL-cholesterol, mmol/L	2.7 ± 0.6	2.7 ± 0.6	0.999
HDL-cholesterol, mmol/L	1.3 ± 0.2	1.0 ± 0.2	< 0.001
CAP, dB/m	N/A	310.6 ± 23.7	N/A
LSM, kPa	N/A	7.6 ± 2.7	N/A

Data are presented as mean ± standard deviation or counts and percentages.

*p*-values were calculated using Student’s *t* test or chi-squared test.

MASLD, metabolic dysfunction-associated steatotic liver disease; BMI, body mass index; PPI, proton pump inhibitor; ALT, alanine transaminase; AST, aspartate aminotransferase; GGT, gamma-glutamyl transferase; CAP, controlled attenuation parameter; LSM, liver stiffness measurement; N/A, not available.

Sequencing of 16S rRNA gene libraries yielded a total of 4,942,037 high-quality reads, with a median of 97,546 reads (range 33,918–124,397) per sample (Supplementary Table S1). Furthermore, almost all rarefaction curves were approaching smoothness with the increase of read number, indicating the sequencing depth was sufficient (Supplementary Fig. S1).

### Alpha- and beta-diversity of the duodenal mucosal microbiota in patients with MASLD

**Figure 1:**
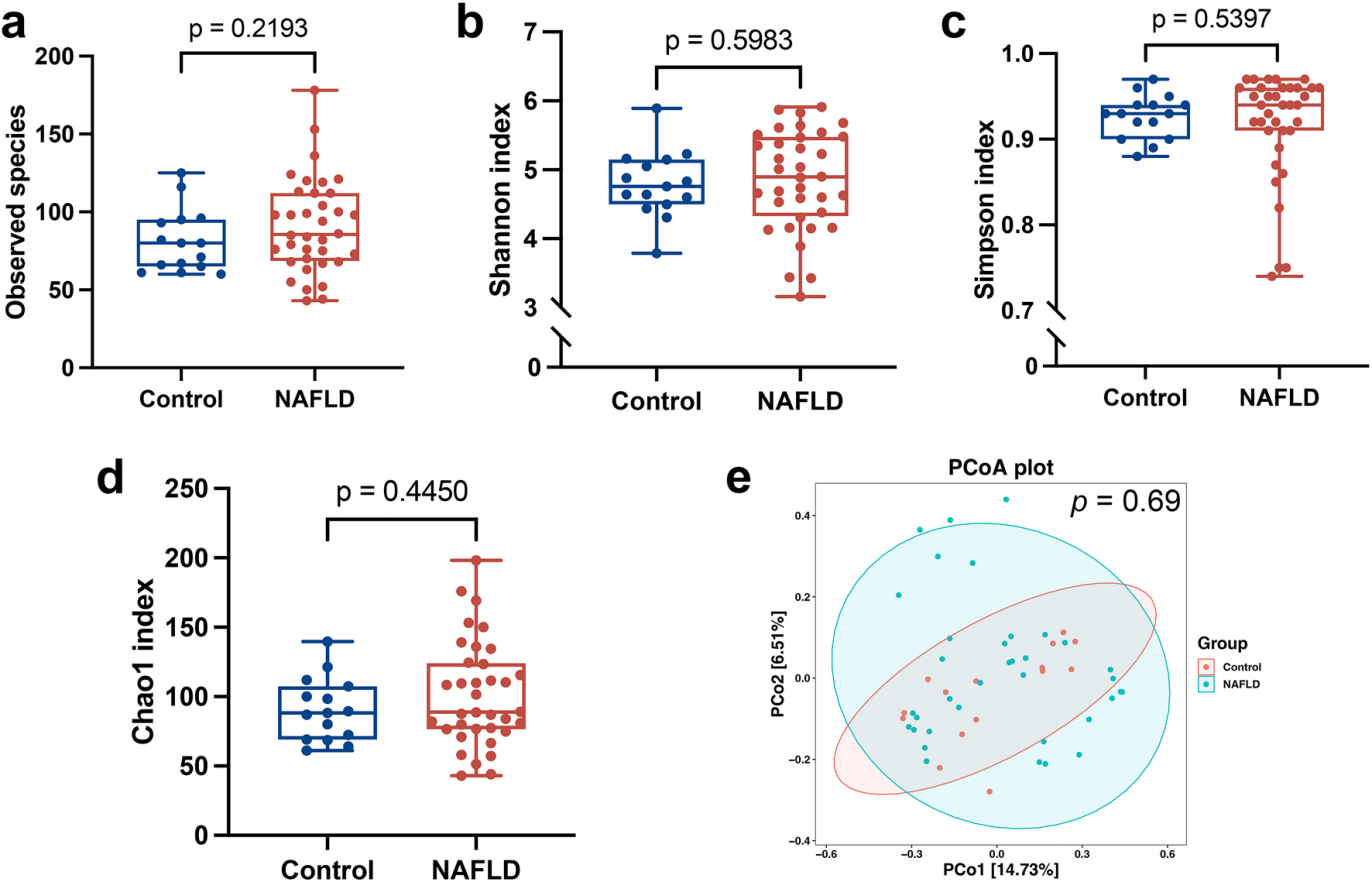
Evaluation of microbial diversity of duodenal mucosal microbiota in patients with MASLD. (**a**) Simpson index, (**b**) Shannon index, (**c**) Chao1 index, and (**d**) observed species were used to assess alpha diversity. (**e**) PCoA was used to evaluate beta diversity based on Bray–Curtis distance. MASLD, metabolic dysfunction-associated steatotic liver disease; PCoA, principal co-ordinate analysis.

Alpha diversity and beta diversity were compared between MASLD subjects and healthy controls to evaluate the characteristics of the duodenal mucosal microbiota associated with MASLD. There was no significant difference in alpha diversity between the two groups (Fig. 1a–d): observed species (*p* = 0.2193, Simpson index (*p* = 0.5983), Shannon index (*p* = 0.5397), and Chao1 index (*p* = 0.4450). Principal co-ordinate analysis (PCoA) was used to examine the extent of similarity of microbial communities based on Bray–Curtis distance, and no significant differences were found between the MASLD group and healthy controls (*p* = 0.69, Fig. 1e).

### Duodenal mucosal microbiome composition at phyla and genera levels

**Figure 2:**
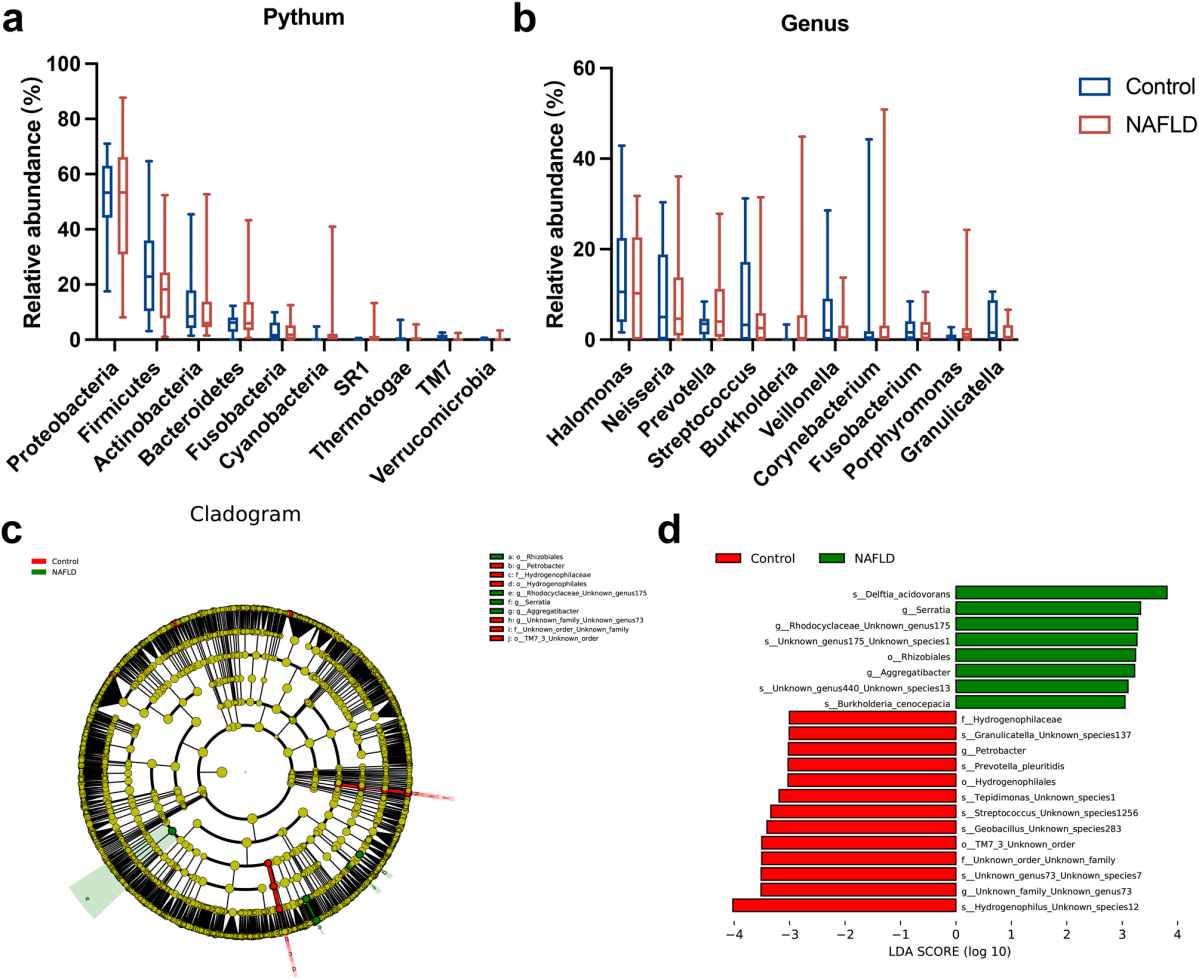
Evaluation of relative abundance of duodenal mucosal microbiota in patients with MASLD. Abundance of microbial taxa in MASLD and healthy controls at phylum (**a**) and genus (**b**) levels. (**c**) Bacterial biomarkers found by linear discriminant analysis effect size (LEfSe). (**d**) Taxonomic cladogram obtained from LEfSe. MASLD, metabolic dysfunction-associated steatotic liver disease.

Approximately 24 bacterial phyla and 526 bacterial genera were detected in the duodenum of patients with MASLD and healthy controls. The duodenal microbiome composition at the phyla level in healthy controls in descending order of abundance was Proteobacteria (50.1%), Firmicutes (26.1%), Actinobacteria (12.2%), Bacteroidetes (5.9%), and Fusobacteria (3.1%), while the five phyla with the highest relative abundance in the MASLD group were Proteobacteria (52.4%), Firmicutes (18.0%), Bacteroidetes (11.3%), Actinobacteria (9.9%), and Fusobacteria (3.1%) (Fig. 2a, Supplementary Table S2).

At the genera level, the five taxa with the highest relative abundance in healthy controls were *Halomonas* (13.8%), *Neisseria* (9.4%), *Streptococcus* (7.2%), *Veillonella* (5.5%), and *Corynebacterium* (3.8%), while in the MASLD group, the five genera with the highest relative abundance were *Halomonas* (11.73%), *Neisseria* (7.32%), *Prevotella* (6.84%), *Burkholderia* (5.87%), and *Streptococcus* (4.48%) (Fig. 2b, Supplementary Table S3).

### Alterations in the composition of the duodenal microbiota associated with MASLD

Linear discriminant analysis (LDA) effect size (LEfSe) analysis was performed to identify specific bacteria associated with MASLD (Fig. 2c and d), which was characterized by higher relative abundances of the genera *Serratia* (LDA = 3.33, *p* < 0.01), *Aggregatibacter* (LDA = 3.22, *p* = 0.03), and *Rhodocyclaceae_Unknown_genus175* levels (LDA = 3.28, *p* = 0.05) (Fig. 2c). Meanwhile, in the healthy control group, the relative abundances of *Petrobacter* (LDA = 3.03, *p* = 0.03) and *Unknown_family_Unknown_genus73* (LDA = 3.51, *p* = 0.02) were significantly increased (Fig. 2c).

### Prediction of the functional profile of the duodenal mucosal microbiome in MASLD

**Figure 3:**
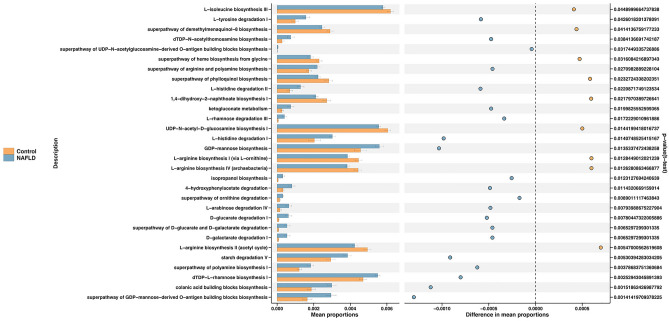
PICRUSt2 prediction of functional composition of duodenal mucosal microbiota in patients with MASLD. MASLD, metabolic dysfunction-associated fatty liver disease.

We applied Phylogenetic Investigation of Communities by Reconstruction of Unobserved States (PICRUSt2) to infer the functional properties of the duodenal microbiota. The results indicated that the duodenal microbiota of the MASLD subjects were characterized by enrichment in degradation pathways especially amino acid degradation (l-tyrosine, l-histidine, and ornithine) and carboxylate degradation, as well as amine and polyamine biosynthesis and sugar nucleotide biosynthesis pathways (Fig. 3). However, healthy controls had a greater abundance of biosynthesis pathways, especially those related to amino acid biosynthesis (l-isoleucine and l-arginine), carbohydrate biosynthesis, and cofactor, carrier, and vitamin biosynthesis (Fig. 3).

### Subgroup analysis of the duodenal mucosal microbiome based on CAP and LSM values

**Figure 4:**
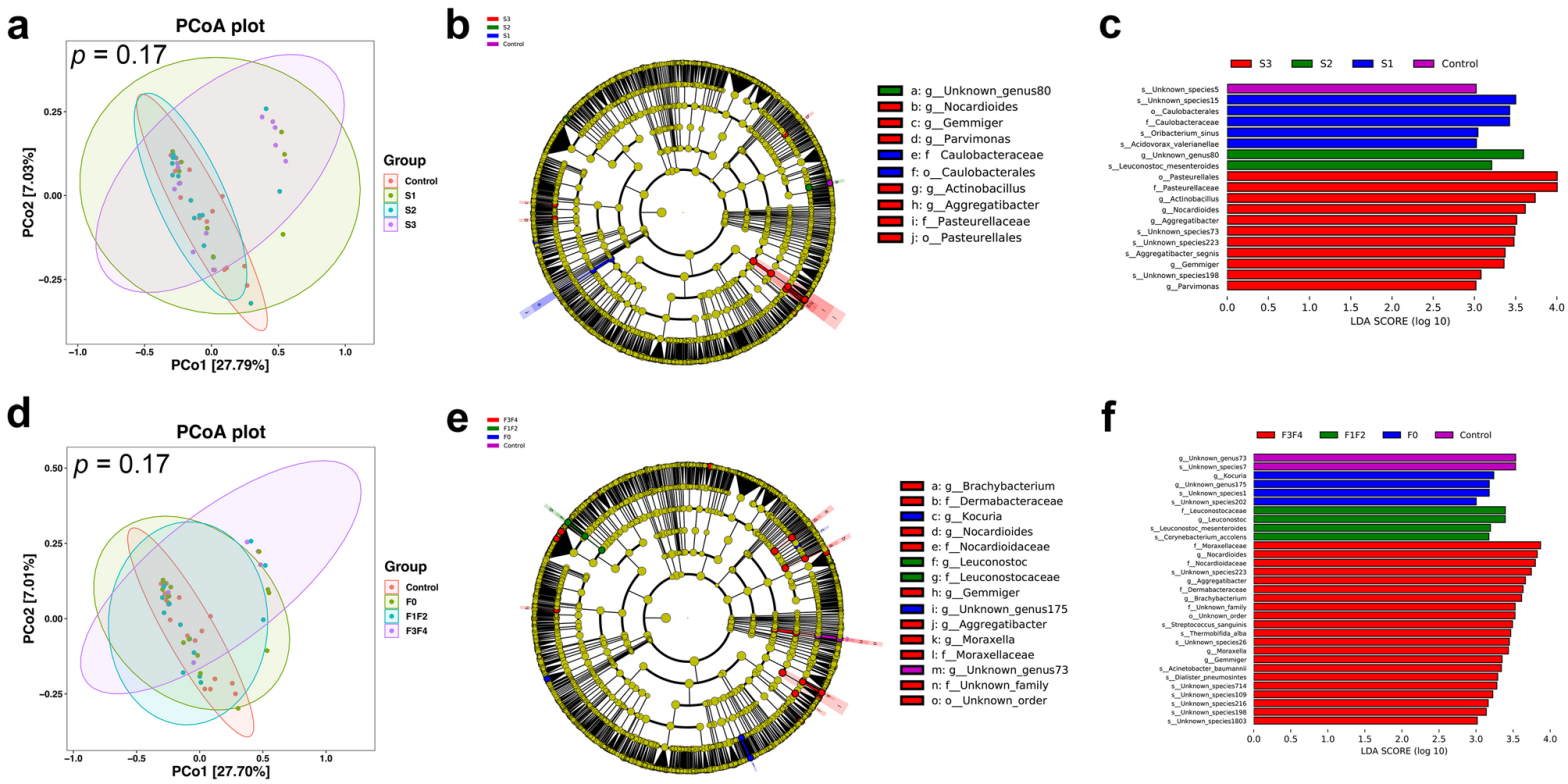
Subgroup analysis of the duodenal mucosal microbiome based on CAP and LSM values. (**a**) PCoA was used to compare beta diversity between subgroups based on CAP values. (**b**, **c**) Bacterial biomarkers subgroups and taxonomic cladogram based on CAP values found by LEfSe analysis. (**d**) PCoA was used to compare beta diversity between subgroups based on LSM values. (**e**,  **f**) Bacterial biomarkers subgroups and taxonomic cladogram based on LSM values found by LEfSe analysis. CAP, controlled attenuation parameter; LSM, liver stiffness measurement; PCoA, principal co-ordinate analysis; LEfSe, linear discriminant analysis effect size.

Based on the CAP and LSM values obtained by transient elastography, patients were categorized as having no steatosis (S0), mild steatosis (S1), moderate steatosis (S2), and severe steatosis (S3), or having no fibrosis (F0), mild to moderate fibrosis (F1–F2) and advanced fibrosis (F3–F4)^20^. Subgroup analysis results showed that there was no significant differences between subgroups in terms of alpha diversity and beta diversity (Fig. 4a and d, Supplementary Fig. S2).

LEfSe analysis revealed that the duodenal microbiome of patients with severe steatosis was characterized by higher relative abundances of the genera *Actinobacillus* (LDA = 3.74, *p* = 0.01), *Nocardioides* (LDA = 3.62, *p* = 0.03), *Aggregatibacter* (LDA = 3.51, *p* = 0.02), *Gemmiger* (LDA = 3.36, *p* = 0.03), and *Parvimonas* (LDA = 3.02, *p* = 0.04) (Fig. 4b and c). Similarly, the duodenal microbiome of patients with advanced fibrosis was characterized by higher relative abundances of the genera *Nocardioides* (LDA = 3.83, *p* = 0.02), *Aggregatibacter* (LDA = 3.67, *p* = 0.04), *Brachybacterium* (LDA = 3.61, *p* < 0.01), *Moraxella* (LDA = 3.44, *p* < 0.01), *Gemmiger* (LDA = 3.35, *p* = 0.02) (Fig. 4e and f).

## Discussion

Dysbiosis of the gut microbiota has been repeatedly observed in MASLD^5,6^. However, previous studies have predominantly focused on feces as these samples are easily accessible and have the highest microbial density of any human sample type. The fecal microbiome represent the large intestine microbiome well but is known to differ substantially from the small intestine microbiome^10^. Therefore, we aimed to characterize the duodenal mucosal microbiome in patients with NFALD compared with healthy controls.

The duodenal ecosystem is characterized by greater motility, lower pH, the presence of bile acid and digestive enzymes, and higher oxygen concentrations compared with the lower gut, which lead to differences in the microbiota between these two regions^21^. Previous studies on duodenal microbiota have sampled various sites within the portions of the duodenum. In addition, some studies sampled duodenal aspirates to investigate luminal microbiota^22,23^, while others sampled mucosal tissue to investigate mucosa-associated microbiota^17,24,25^. Recently, Constante et al.^26^ reported that gut location is a key determinant of duodenal microbiota composition and predicted function in celiac disease. In the present study, Proteobacteria and Firmicutes were the two major phyla in the mucosa of the descending duodenum in both groups. Compared with duodenal luminal microbiota where Firmicutes and Bacteroidetes are the two major phyla^22,23^, the findings from the current study were consistent with previous studies that Proteobacteria and Firmicutes are major phyla in the duodenal mucosa^17,24^. However, some previous studies on duodenal mucosa-associated microbiota have reported Firmicutes to be the phylum with the highest relative abundance^25,27^, which is inconsistent with the results of the present study. Relatively few studies have been conducted on the small intestinal microbiota to date, and additional research in this area is needed in the future to determine the microbial profile of the small intestine.

The duodenum and its microbes are increasingly implicated in the pathophysiology of metabolic diseases^28^. Alterations of the duodenal microbiome have been associated with obesity^29,30^, hyperglycemia^31^, and type 2 diabetes^32^. Duodenal delivery of fecal microbiota transplantation from lean donors increased insulin sensitivity in individuals with metabolic syndrome^33^. Recently, Koopen et al.^34^ found that duodenal single dose infusion of *Anaerobutyricum soehngenii* stimulated glucagon-like peptide-1 (GLP-1) production, ameliorated glycemic control and beneficially shaped the duodenal transcriptome in subjects with metabolic syndrome. Since MASLD is associated with various disease conditions including obesity, insulin resistance, diabetes, hypertension, and hyperlipidemia, and is considered a hepatic manifestation of metabolic syndrome^35^, it is likely that the duodenal microbiota are associated with the pathogenesis of MASLD. Consequently, targeting the duodenal microbiota may provide therapeutic opportunities for MASLD.

In the present study, medication was not an exclusion criterion. Although no differences in gut microbiota between MASLD and healthy individuals as a whole were observed in the current study, key members of the microbiota could be identified. LEfSe analysis found that patients with MASLD were characterized by a dominance shift toward specific potentially pathogenic bacterial genera (*Serratia* and *Aggregatibacter*), while the genus *Petrobacter* was enriched in healthy controls. A higher relative abundance of the genus *Streptococcus* was reported in the duodenal microbiota of subjects with alcoholic liver disease^18^ and chronic liver disease^19^. However, in the present study, there was no significant difference in the relative abundance of *Streptococcus* between patients with MASLD and healthy controls. In cirrhotic patients, the relative abundances of *Veillonella*, *Megasphaera*, *Dialister*, *Atopobium*, and *Prevotella* were increased in the duodenal mucosa^15^; however, none of these genera were significantly altered in patients with MASLD in the current study.

The enriched pathways in MASLD patients were related to degradation especially amino acid degradation, while pathways related to biosynthesis especially amino acid biosynthesis were enriched in healthy subjects. The most abundant amino acid fermenting bacteria in the human small intestine have been reported to be *Clostridium*, the *Bacillus-Lactobacillus-Streptococcus* groups, and *Proteobacteria*^36^. Moreover, different dietary habits (omnivorous, vegetarians, and vegans) can influence the microbial functions in humans^37^. Therefore, we speculate that the altered microbial function is due to differences in community structures and different dietary habits. Further robust metagenomics and metabolomics analyses are needed to interrogate this relationship more comprehensively.

Aging^38^, smoking^39^, PPI use^40^, and *H. pylori* infection^23,24^ are known to affect duodenal microbiota. However, in the present study, none of these variables affected the alpha- or beta-diversity of duodenal microbiota (Supplementary Fig. S3 and S4), possibly due to the small sample size. Moreover, no significant correlation were found between clinical variables (BMI, ALT, AST, GGT, triglycerides, total cholesterol, CAP, or LSM) and microbial diversity or relative abundance of any significantly altered microbial genus (all *p* value > 0.05; Supplementary Fig. S5). Studies with larger sample sizes are required to elucidate the association between clinical features and duodenal mucosal microbiota in patients with MASLD.

There are several limitations to the present study. First, most of the enrolled patients with MASLD were obese, and the BMI of the MASLD group was significantly higher compared with that of the healthy control group. This may have affected the results of this study, as previous studies have shown that the duodenal microbiota is altered in obese patients compared with individuals with normal BMI^29,30^. However, it seems impossible to distinguish MASLD from obesity, as MASLD is a metabolic disease at the interface of obesity, metabolic syndrome, and type 2 diabetes. Second, the healthy controls in the current study were undergoing gastroscopy to assess intestinal discomfort, including dyspepsia and gastroesophageal reflux disease, as well as for screening purposes due to associated risk factors. Therefore, these subjects may not fully represent normal healthy individuals, and the medications they take (especially PPIs, H2 receptor antagonists, prokinetic drugs, etc*.*) may have affected the duodenal microbiota. Furthermore, predicting microbial functional genes based on their taxonomic composition using PICRUSt2 is unreliable to some extent^41^, nevertheless, the better method shotgun metagenome sequencing is limited by domination of non-microbial DNA in duodenal mucosa specimens. Finally, traditional disposable biopsy forceps were used to sample duodenal mucosal tissue in this study, although new device is available that allows sampling of the duodenal mucosa without contamination from luminal contents or other regions of the gastrointestinal tract^39^.

Despite these limitations, the current study does have several strengths. For the first time, the duodenal mucosal microbiome profile was characterized in patients with MASLD. Five regions of the 16S rRNA gene were amplified and sequenced to maximize detection of potential microbiota in the duodenal mucosa. Moreover, negative controls were established during sampling, DNA extraction, and PCR amplification to reduce potential contamination during the process.

In conclusion, the findings of this study reveal the duodenal mucosal microbiota in patients with MASLD. Further studies are needed to establish a causal relationship between duodenal microbiota and MASLD.

## Materials and methods

### Study population

Patients with MASLD as well as healthy control subjects aged 18–75 years undergoing routine gastroscopy examination were included in the study. Subjects were recruited at the department of gastroenterology, the first affiliated hospital, Zhejiang university school of medicine, from September 2020 to April 2022. MASLD was defined as hepatic steatosis along with at least 1 of 5 metabolic risk factors (obesity, hyperglycemia, elevated blood pressure, increased plasma triglycerides, and decreased HDL-cholesterol)^42^. Healthy control subjects were determined to have no hepatic steatosis by abdominal ultrasonography. Patients were excluded if they had taken antibiotics, prebiotics, or probiotics in the four weeks prior to endoscopy; if they exhibited evidence of organic gastrointestinal diseases based on endoscopic and clinical histological findings (other than non-erosive reflux disease); and if they received bowel preparation before endoscopy. Informed consent was obtained from all participants and ethical approval was granted by the ethics committee of the first affiliated hospital, Zhejiang university school of medicine (approval number 2021ITT613). The study was performed in accordance with the principles of the Declaration of Helsinki.

LSM and CAP were obtained by transient elastography using the FibroScan PRO (Echosens, Paris, France) as previously described^43^. BMI, liver enzymes (ALT, AST, and GGT), lipid profile (triglycerides, total cholesterol, LDL-cholesterol, and HDL-cholesterol) and fasting blood glucose were also evaluated.

### Sample collection

Two intestinal biopsies per patient were collected from the descending portion of the duodenum opposite the ampulla of Vater using sterile disposable biopsy forceps (Micro-Tech, Nanjing, China) and were placed into 2-mL sterile tubes. Potential contamination during sampling was excluded through the use of sampling controls where the sterile tubes were opened while biopsies were performed but no biopsy tissue was placed in them^44^. Both biopsy samples and sampling controls were immediately flash frozen in liquid nitrogen and then stored at − 80 °C.

### DNA extraction

DNA was extracted from duodenal tissues and sampling controls using a modified hexadecyltrimethylammonium bromide (CTAB) method^45^. Each sample was placed in a sterile EP tube containing 1 × 1 mL CTAB butter (0.1 M Tris–HCl pH 8.0, 20 mM EDTA pH 8.0, 1.4 M NaCl, 2% CTAB; Zeheng, Hangzhou, China), 2.5 mg lysozyme (Solarbio, Beijing, China), and 0.4 mg proteinase K (Yeasen, Shanghai, China). Homogenization was undertaken in a tissue homogenizer for 10 min followed by incubation at 60 °C for 20 min and 70 °C for 25 min. The lysate from each sample was collected and centrifuged at 8000 g for 5 min at room temperature (RT), then the supernatant was mixed thoroughly with chloroform. After centrifugation at 13,000 g for 10 min at RT, the supernatant was collected and DNA in the supernatant was quantified by Qubit (Thermo Fisher) and stored at − 20 °C.

It was reported that DNA contamination during processing and in the reagents used can yield spurious results in samples with relatively low microbial biomass^46^. Therefore, to exclude potential contamination, negative controls such as DNA extraction controls and no-template polymerase chain reaction (PCR) amplification controls were included in the study.

### Library preparation and sequencing

Library preparation was performed according to a previous study^44^. Briefly, five regions of the 16S rRNA gene were amplified using a set of 10 multiplexed primers (all 5′–3′; F1-TGGCGAACGGGTGAGTAA, F2-ACTCCTACGGGAGGCAGC, F3-GTGTAGCGGTGRAATGCG, F4-GGAGCATGTGGWTTAATTCGA, F5-GGAGGAAGGTGGGGATGAC, R1-AGACGTGTGCTCTTCCGATCTCCGTGTCTCAGTCCCARTG, R2-AGACGTGTGCTCTTCCGATCTGTATTACCGCGGCTGCTG, R3-AGACGTGTGCTCTTCCGATCTCCCGTCAATTCMTTTGAGTT, R4-AGACGTGTGCTCTTCCGATCTCGTTGCGGGACTTAACCC, R5-AGACGTGTGCTCTTCCGATCTAAGGCCCGGGAACGTATT). Amplification was conducted using the Phusion Hot Start Flex 2 × Master Mix (NEB) according to the manufacturer’s instructions. Through a second PCR, barcodes and Illumina adaptors were added to the amplicons. The resulting PCR products were purified using AMPure XT beads (Beckman Coulter Genomics, Danvers, MA, USA). The libraries were then sequenced on an Illumina NovaSeq 6000 system using the NovaSeq 6000 SP Reagent Kit (500 cycles).

### Bioinformatics

The Short MUltiple Regions Framework (SMURF) was applied to combine sequencing results from the five regions of the 16S rRNA gene into a coherent solution^47^. The Silva database version 138 was used as a reference database^48^. Samples with fewer than 1000 normalized reads (including negative controls) and species with relative abundances of < 10^–4^ were discounted from further analysis. Microorganisms with > 50% prevalence in the negative controls (sampling controls, DNA extraction controls, and no-template PCR amplification controls) were identified as contaminating microbiota. A series of six filters were subsequently applied to remove contaminating microbiota according to a previous study^44^, and bioinformatics analyses were performed on the remaining microbiota in the samples. Counts were normalized by dividing reads by the total number of reads in the corresponding sample (Supplementary Table S1).

### Statistical analyses

Clinical variables were presented as mean and standard deviation or counts and percentages and were analyzed with Student’s *t*-test or chi-squared test where appropriate. Spearman correlation was used for evaluating correlations. Statistical significance was defined as *p* < 0.05. Statistical analyses were performed using Statistical Package for Social Sciences (SPSS) version 26.0 (IBM Corporation, Armonk, NY, USA).

Microbiome bioinformatics were performed with QIIME 2 and R software version 3.4.4. Alpha diversity was assessed using the Shannon index, Simpson index, Chao1 index, and observed species, and differences in alpha diversity between groups were compared using the Mann–Whitney U test or Kruskal–Wallis test. Beta diversity was assessed by PCoA and analysis of similarity (ANOSIM), applying the Bray–Curtis distance metric. LEfSe and Mann–Whitney U test were used to identify taxa associated with MASLD^49^. Functional profiles of microbial communities were predicted using Phylogenetic Investigation of Communities by Reconstruction of Unobserved States (PICRUSt2)^50^, and examination of statistical differences was performed using the Statistical Analysis of Metagenomic Profiles (STAMP) software package^51^.

### Supplementary Information

Supplementary Figures.

Supplementary Table S1.

Supplementary Table S2.

Supplementary Table S3.

## Data Availability

The datasets generated during and/or analyzed during the current study are available from the corresponding author on reasonable request.
