# Supplementary material for: Alterations of the duodenal mucosal microbiome in patients with metabolic dysfunction-associated steatotic liver disease
Source: Sci Rep. 2024 Apr 21;14:9124. doi: 10.1038/s41598-024-59605-3 (PMC11032335; doi:10.1038/s41598-024-59605-3)
Supplement: Supplementary file 1 — Supplementary Figures. [file 41598_2024_59605_MOESM1_ESM.pdf]

Alterations of the duodenal mucosal microbiome in patients with metabolic dysfunction-associated steatotic liver disease

Mengting Ren[1,2#], Hanghai Pan[1,2#], Xinxin Zhou[1], Mosang Yu[1], Feng Ji[1*]

1 Department of Gastroenterology, The First Affiliated Hospital, Zhejiang University School of Medicine, Hangzhou, Zhejiang, China

2 Cancer Center, Department of Gastroenterology, Zhejiang Provincial People's Hospital (Affiliated People's Hospital), Hangzhou Medical College, Hangzhou, Zhejiang, China

#Contributed equally.

***Correspondence author**

Feng Ji, MD

Department of Gastroenterology

The First Affiliated Hospital, Zhejiang University School of Medicine

79 Qingchun Road, Hangzhou, Zhejiang, 310003, China

Telephone: +86-571-87236863, Fax: +86-571-87236611

E-mail: jifeng@zju.edu.cn

Supplementary Figures

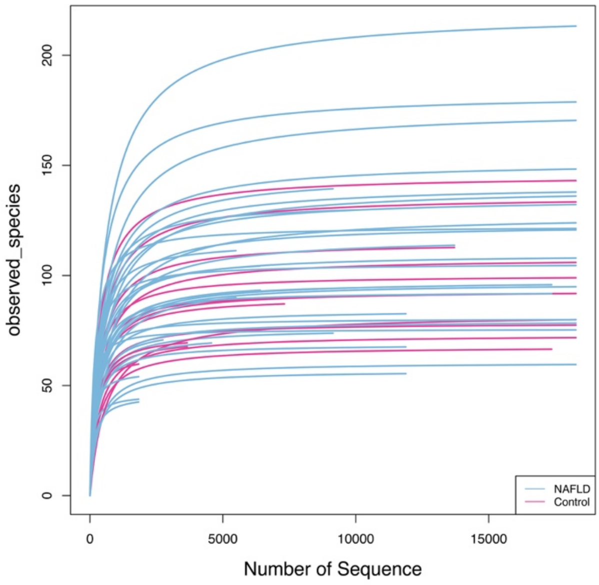

Supplementary Fig. S1. Rarefaction curves of duodenal mucosal samples from MASLD and healthy controls. MASLD, metabolic dysfunction-associated steatotic liver disease.

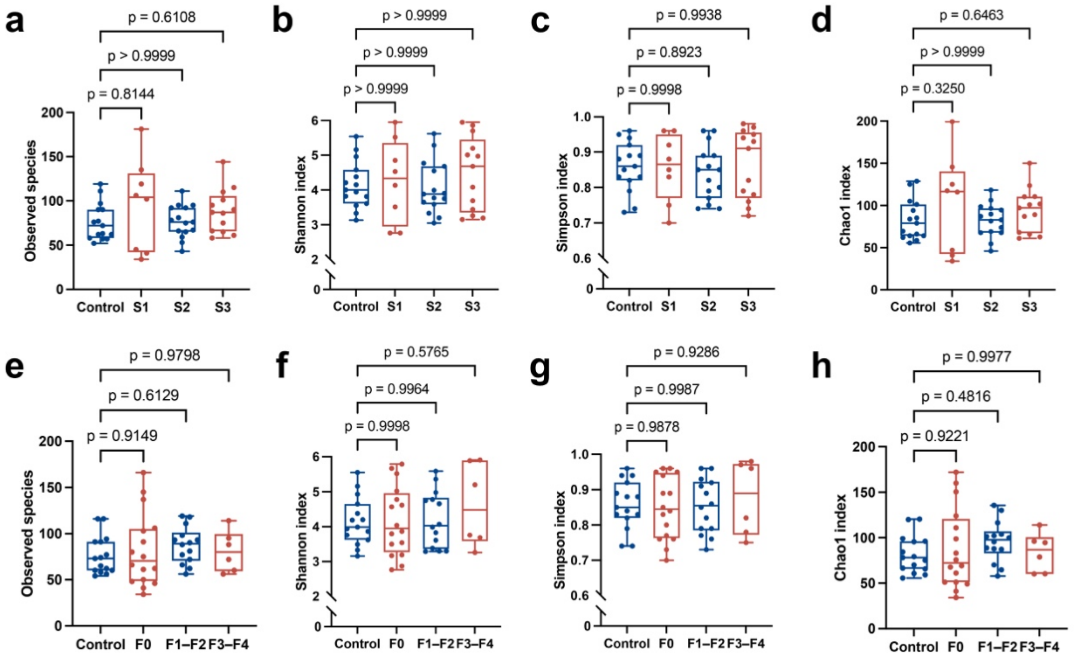

Supplementary Fig. S2. Subgroup analysis of alpha diversity in the duodenal mucosal microbiome based on CAP and LSM values. (a) Simpson index, (b) Shannon index, (c) Chao1 index, and (d) observed species of subgroups based on CAP values. (e) Simpson index, (f) Shannon index, (g) Chao1 index, and (h) observed species of subgroups based on LSM values. CAP, controlled attenuation parameter; LSM, liver stiffness measurement.

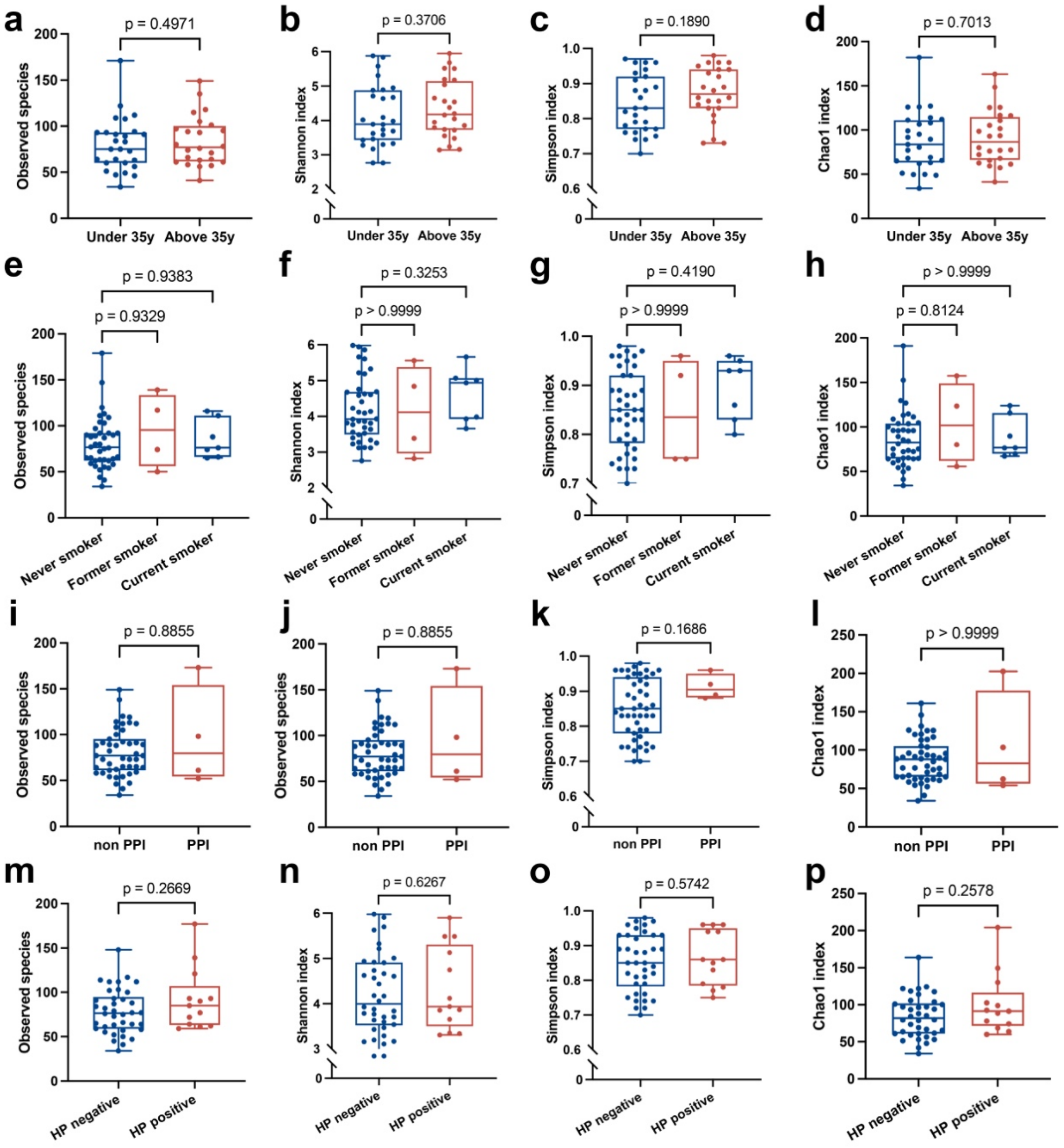

Supplementary Fig. S3. Subgroup analysis of alpha diversity in the duodenal mucosal microbiome based on age, smoking status, PPI use, and *H. Pylori* infection. (a) Simpson index, (b) Shannon index, (c) Chao1 index, and (d) observed species of subgroups based on age. (e) Simpson index, (f) Shannon index, (g) Chao1 index, and (h) observed species of subgroups based on smoking status. (i) Simpson index, (j) Shannon index, (k) Chao1 index, and (l) observed species of subgroups based on PPI use. (m) Simpson index, (n) Shannon index, (o) Chao1 index, and (p) observed species of subgroups based on *H. Pylori* infection. PPI, proton pump inhibitor.

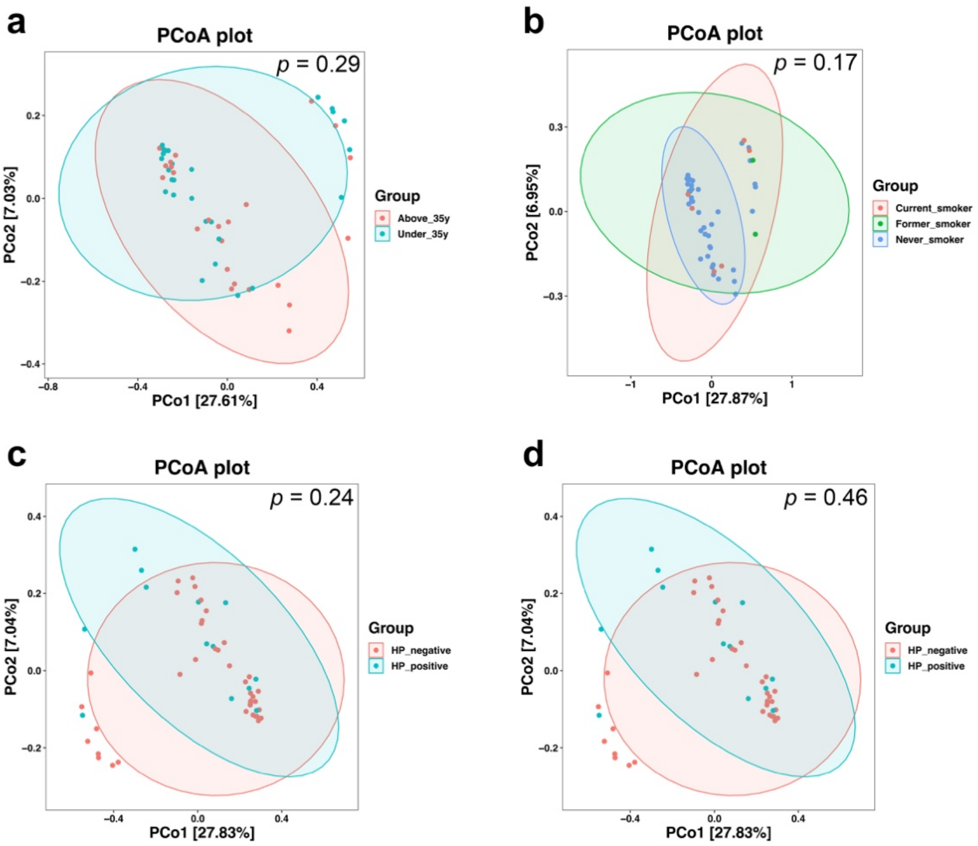

Supplementary Fig. S4. Subgroup analysis of beta diversity in the duodenal mucosal microbiome based on age, smoking status, PPI use, and *H. Pylori* infection. PCoA was used to compare beta diversity between subgroups based on (a) age, (b) smoking status, (c) PPI use, and (d) *H. Pylori* infection. PPI, proton pump inhibitor; PCoA, principal co-ordinate analysis.

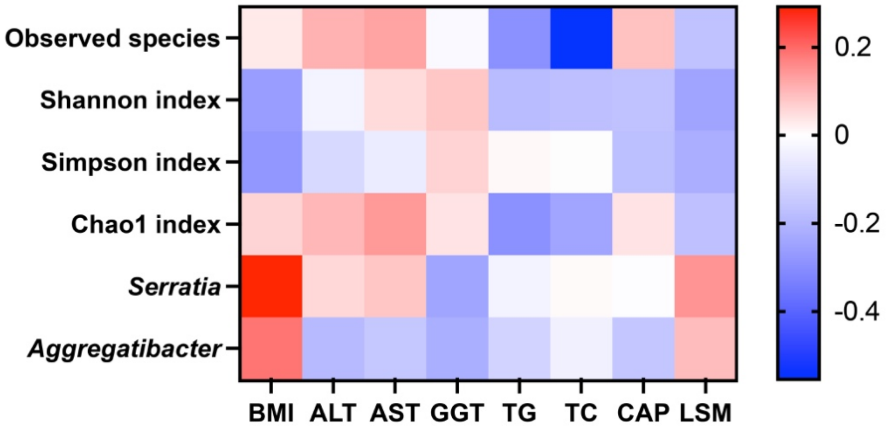

Supplementary Fig. S5. Heatmap showing Spearman correlation coefficients between clinical variables (BMI, ALT, AST, GGT, triglycerides, total cholesterol, CAP, or LSM) and microbial diversity or relative abundance of any significantly altered microbial genus. All p values > 0.05. BMI, body mass index; ALT, alanine transaminase; AST, aspartate aminotransferase; GGT, gamma-glutamyl transferase; CAP, controlled attenuation parameter; LSM, liver stiffness measurement.
